# MusicARLtrans Net: a multimodal agent interactive music education system driven via reinforcement learning

**DOI:** 10.3389/fnbot.2024.1479694

**Published:** 2024-11-21

**Authors:** Jie Chang, Zhenmeng Wang, Chao Yan

**Affiliations:** ^1^School of Music, Sangmyung University, Seoul, Republic of Korea; ^2^School of Music, Qufu Normal University, Rizhao, China; ^3^School of Computer Science, Qufu Normal University, Rizhao, China

**Keywords:** Speech-to-Text, ALBEF, reinforcement learning, multimodal agent, music speech recognition

## Abstract

**Introduction:**

In recent years, with the rapid development of artificial intelligence technology, the field of music education has begun to explore new teaching models. Traditional music education research methods have primarily focused on single-modal studies such as note recognition and instrument performance techniques, often overlooking the importance of multimodal data integration and interactive teaching. Existing methods often struggle with handling multimodal data effectively, unable to fully utilize visual, auditory, and textual information for comprehensive analysis, which limits the effectiveness of teaching.

**Methods:**

To address these challenges, this project introduces MusicARLtrans Net, a multimodal interactive music education agent system driven by reinforcement learning. The system integrates Speech-to-Text (STT) technology to achieve accurate transcription of user voice commands, utilizes the ALBEF (Align Before Fuse) model for aligning and integrating multimodal data, and applies reinforcement learning to optimize teaching strategies.

**Results and discussion:**

This approach provides a personalized and real-time feedback interactive learning experience by effectively combining auditory, visual, and textual information. The system collects and annotates multimodal data related to music education, trains and integrates various modules, and ultimately delivers an efficient and intelligent music education agent. Experimental results demonstrate that MusicARLtrans Net significantly outperforms traditional methods, achieving an accuracy of **96.77%** on the LibriSpeech dataset and **97.55%** on the MS COCO dataset, with marked improvements in recall, F1 score, and AUC metrics. These results highlight the system's superiority in speech recognition accuracy, multimodal data understanding, and teaching strategy optimization, which together lead to enhanced learning outcomes and user satisfaction. The findings hold substantial academic and practical significance, demonstrating the potential of advanced AI-driven systems in revolutionizing music education.

## 1 Introduction

The technology of music voice recognition holds extensive application potential not only in areas such as music information retrieval, music education, and digital entertainment but also plays a crucial role in the development of intelligent voice assistants and accessibility technologies. With continuous advancements in artificial intelligence and machine learning, music voice recognition can offer users more accurate music search and recommendation systems, as well as support in music creation and learning. However, the complexity of music voice recognition far surpasses that of general voice recognition due to the need for multi-dimensional analysis, including pitch, rhythm, and timbre. Therefore, in-depth research into music voice recognition is essential, not only to enhance the accuracy of current technologies but also to foster innovation and development in related fields.

Traditional methods for music voice recognition primarily involve symbolic AI and knowledge representation. Among these, expert systems were early technologies that relied on predefined rules and knowledge bases to process musical information. For instance, some systems perform music analysis using rules based on music theory (Exposito et al., 2006), while others employ expert systems for automated score analysis (Betancourt et al., 2018). Another approach is rule-based systems, which utilize detailed music rules for recognition and parsing, such as systems based on pitch and rhythm rules (Oramas et al., 2015) or systems combining note and rhythm pattern rules (Liu and Fung, 2000). Additionally, gradient-based methods, as a more recent technology, enhance the recognition process through optimization algorithms and gradient descent, including gradient optimization methods using convolutional neural networks (Costa et al., 2017) and advanced deep learning algorithms (Hongdan et al., 2022). These methods are theoretically robust and interpretable, enabling them to manage complex music patterns and rules to a certain extent. However, they often struggle with adaptability to complex musical variations, particularly when dealing with non-standard music data or rapidly changing musical features, which can lead to reduced recognition accuracy.

To overcome the limitations of traditional algorithms in handling complex music data, data-driven and machine learning algorithms have increasingly been applied to music voice recognition. These methods mainly include decision trees, random forests, and multi-layer perceptrons (MLPs). The decision tree algorithm classifies and predicts music data by creating a tree-like model that recursively splits the data into different categories, clearly illustrating the relationships between features (Lavner and Ruinskiy, 2009). Another commonly used method is random forests, which enhance classification accuracy by combining multiple decision trees. Each tree is trained on a random subset of the data and features, and the final prediction is determined by a vote among these trees, effectively reducing overfitting and increasing model stability (Thambi et al., 2014). Additionally, multi-layer perceptrons (MLPs), a deep learning approach, learn complex music features through a multi-layer neural network structure. Each layer's neurons transform the input data using nonlinear activation functions, enabling the model to capture high-level features and thus improve recognition accuracy (Ajmera et al., 2003). While these methods offer significant flexibility and robustness in handling complex music features and patterns, they also face challenges related to the high computational complexity of large-scale data and high-dimensional features, which can lead to prolonged training times and considerable computational resource demands.

As a solution to the limitations of traditional statistical and machine learning algorithms in handling complex music features, deep learning algorithms have become increasingly dominant in music voice recognition. These methods primarily include Convolutional Neural Networks (CNNs), Reinforcement Learning, Transformers, and Attention Mechanisms. Convolutional Neural Networks (CNNs) effectively extract spatial and temporal features from music signals through multiple layers of convolution and pooling operations, demonstrating superior performance in audio classification and feature extraction tasks (Hema and Marquez, 2023). Reinforcement Learning optimizes models through interactions with the environment, enabling adaptive learning and improvement of strategies, thus exhibiting strong self-learning capabilities in music generation and real-time adjustment tasks (Chen et al., 2023). Transformer models, which use self-attention mechanisms to model input sequences, capture long-range dependencies and have significant advantages in modeling and generating music sequences (Wen and Zhu, 2022). Attention Mechanisms dynamically adjust the weights of input data, enhancing the ability to capture important features and have achieved notable success in music translation and generation tasks (Li et al., 2021). While these methods offer powerful feature learning and modeling capabilities that significantly improve recognition and generation accuracy, they come with high computational resource demands, lengthy training times, and increased model complexity, which can substantially raise computational costs and training challenges, especially when handling large-scale datasets.

To address the high computational resource demands, extended training times, and model complexity associated with these methods for handling complex music features, we propose a novel solution: MusicARLtrans Net. This multi-modal intelligent interactive music education system is designed to overcome the limitations of traditional deep learning methods through reinforcement learning. MusicARLtrans Net combines reinforcement learning with multi-modal inputs, dynamically adjusting and optimizing the music learning process through interactions between intelligent agents and users. The motivation behind this approach is to enhance the personalization and interactivity of music education while effectively reducing reliance on computational resources. By leveraging reinforcement learning, the system can adaptively adjust teaching strategies and provide personalized feedback, thereby improving the effectiveness and efficiency of music education. Additionally, the use of multi-modal inputs allows the system to integrate audio, visual, and textual data, making it more comprehensive and precise when handling complex music data. Through this approach, we aim to achieve efficient learning while reducing the computational complexity and time consumption associated with traditional methods.

- MusicARLtrans Net introduces a novel combination of reinforcement learning and multi-modal inputs, innovatively applying an intelligent agent system to music education. By leveraging adaptive learning and interactive optimization, the system enhances educational outcomes.
- The system features multi-scenario adaptability, efficiently handling audio, visual, and textual data. It achieves personalization and versatility in music education, making it suitable for various learning environments and needs.
- Experiments show that MusicARLtrans Net outperforms traditional methods in music learning tasks, significantly improving learning efficiency and accuracy while reducing computational resource demands.

## 2 Related work

### 2.1 Speech recognition

The development of speech recognition technology has evolved over several decades, transitioning from early rule-based systems to modern deep learning models through multiple pivotal stages. Initially, speech recognition systems relied on expert systems and rule-based approaches, which typically featured limited vocabularies and lower accuracy. However, the introduction of statistical learning methods, particularly the application of Hidden Markov Models (HMMs) and Gaussian Mixture Models (GMMs), marked significant advancements in the 1980s and 1990s. HMM-GMM models effectively captured the temporal characteristics of speech, greatly enhancing recognition accuracy (Wang et al., 2019b). As the 21st century unfolded, the rise of machine learning, particularly deep learning, further accelerated the progress of speech recognition technology. Models based on Deep Neural Networks (DNNs), Convolutional Neural Networks (CNNs), and Long Short-Term Memory Networks (LSTMs) have demonstrated remarkable performance in speech recognition tasks. These deep learning models can automatically extract features from speech signals, significantly reducing the need for manual feature engineering and improving the accuracy and robustness of speech recognition systems (Lin et al., 2024b). In recent years, end-to-end speech recognition models, such as Deep Speech and Transformer-based architectures like Google's WaveNet and OpenAI's Whisper, have further streamlined the speech recognition process while achieving notable improvements in system performance. These models integrate all components of speech recognition into a single neural network, simplifying the architecture and enhancing overall efficiency (Lin et al., 2024a). Moreover, the application scope of speech recognition technology has expanded considerably—from early implementations in telephone customer service systems and voice assistants to today's smart homes, automotive voice control systems, and healthcare monitoring devices. Speech recognition technology holds immense potential, particularly in areas such as multilingual and dialect recognition, robustness in noisy environments, and real-time speech translation. As more advanced deep learning algorithms and larger datasets become available, speech recognition technology is poised to deliver more natural, precise, and versatile applications.

### 2.2 Robotic vision

Robotic vision is a pivotal research area within artificial intelligence and robotics, focused on equipping robots with the ability to comprehend and interpret visual information. The evolution of robotic vision has transitioned from traditional image processing and computer vision techniques to modern deep learning methods, progressing through several significant stages. Early robotic vision systems primarily relied on classical image processing techniques, such as edge detection, shape recognition, and feature extraction. While these methods were somewhat effective for simple visual tasks, they often struggled in complex environments (Wang et al., 2016). With the dawn of the 21st century, advancements in computing power and the accumulation of big data catalyzed significant breakthroughs in robotic vision technology, particularly through the adoption of machine learning and deep learning techniques. Convolutional Neural Networks (CNNs) have become central to this field, as they can automatically extract multi-level features from images through multiple layers of convolution and pooling operations. This approach has greatly enhanced performance in image classification, object detection, and semantic segmentation. Notable deep learning models, such as AlexNet, VGGNet, ResNet, and YOLO, have propelled robotic vision into a new era, demonstrating exceptional performance on large datasets like ImageNet (Hong et al., 2024). Beyond the understanding of static images, robotic vision must also process information from dynamic scenes, requiring technologies like video analysis and 3D reconstruction. Optical flow methods and object tracking algorithms analyze motion information in videos, while structured light, stereo vision, and SLAM (Simultaneous Localization and Mapping) technologies are employed to construct 3D models of the environment. The integration of these technologies enables robots to navigate, recognize objects, and interact with humans in complex and dynamic settings. In recent years, the integration of multimodal information in robotic vision has gained increasing attention. By combining visual, auditory, and tactile modalities, this approach enhances a robot's ability to understand its environment and execute tasks more effectively. As more advanced deep learning algorithms and sensor technologies continue to develop, robotic vision is expected to achieve even greater precision, robustness, and real-time performance. These advancements will drive extensive applications across various sectors, including manufacturing, service industries, healthcare, and beyond (Fishel and Loeb, 2012).

### 2.3 Reinforcement learning

Reinforcement Learning (RL) is a machine learning approach that enables agents to learn optimal actions within an environment by maximizing cumulative rewards through a trial-and-error process. The theoretical underpinnings of RL are rooted in Markov Decision Processes (MDP) and dynamic programming. However, significant advancements in computational power and the integration of deep learning have propelled RL to new heights, enabling its application across a wide range of practical domains. In the realm of gaming, RL has demonstrated remarkable potential. A notable example is DeepMind's AlphaGo, which defeated the world champion in Go using deep reinforcement learning, marking a significant milestone in the field. This achievement highlighted RL's potential in mastering complex strategic games. Building on this success, AlphaZero further showcased RL's generalization capabilities by combining self-play with deep neural networks, excelling not only in Go but also in chess and shogi (Wang et al., 2019a). In robotics, RL has proven effective in learning optimal control strategies for robots tasked with complex operations. Traditional robot control methods often rely on predefined rules and models, whereas RL enables robots to autonomously learn optimal action strategies through interaction with their environment. This approach has been widely applied in areas such as robotic arm manipulation, autonomous drone flight, and path planning for self-driving cars. The integration of deep reinforcement learning allows robots to master complex control strategies within high-dimensional, continuous action spaces (Oudeyer and Kaplan, 2007). The financial sector represents another significant application area for RL. By modeling financial environments such as stock markets, RL assists in designing and optimizing trading strategies, asset allocation, and risk management. RL algorithms learn market dynamics and adapt autonomously to varying market conditions, enhancing the accuracy and profitability of investment decisions. In natural language processing (NLP) and dialogue systems, RL is employed to optimize dialogue strategies and generate natural language responses. For instance, RL can empower chatbots to learn how to guide users through conversations, offer personalized recommendations, and improve overall user satisfaction. Additionally, RL is leveraged to optimize advertising strategies by dynamically adjusting ad content and timing in real-time, thereby maximizing user click-through rates and conversion rates (Ai et al., 2023).

## 3 Methodology

### 3.1 Overview of our network

Our approach centers on adapting the ALBEF (Align Before Fuse) multimodal architecture to better cater to the specific needs of music education through voice interactions. Traditionally, ALBEF fuses visual and textual modalities to enable a comprehensive understanding of inputs. In our system, we replace the visual editor with an audio editor to align with the primary task of speech recognition. This adaptation allows us to focus on enhancing audio processing capabilities, making the system more effective for music education.

A key innovation in our approach is the development of a novel Speech-to-Text (STT) model specifically tailored for the music education domain. This model integrates a new Acoustic Model (AM) and Language Model (LM). The AM is designed to capture the nuances of musical terminology and speech patterns relevant to music education, while the LM facilitates accurate transcription of domain-specific language. By combining these models, we significantly improve the system's ability to transcribe and interpret user inputs with precision. Furthermore, to align with our audio editor, we implemented a refined Transformer-based text editor. This editor reduces the original 6-layer Transformer structure to a more efficient 3-layer configuration, enhancing processing speed while maintaining high effectiveness.

The implementation of our music education system involves several key steps, each designed to optimize the integration of the audio editor, STT model, and refined text editor, with reinforcement learning enhancing the overall performance. 1. Audio editor and STT model development: The first step involves developing and training the Speech-to-Text model. The Acoustic Model (AM) is meticulously designed to process and interpret musical terms and speech patterns. This model is trained using a comprehensive dataset that includes various musical and educational audio samples, ensuring it captures the specific characteristics of the music domain. Concurrently, the Language Model (LM) is developed to understand and generate domain-specific phrases, improving the accuracy of text transcriptions related to music education. The STT model is then integrated into the audio editor, which is tailored to handle the complexities of music-related speech. 2. Adaptation of text editor: The text editor, essential for processing the transcribed text from the STT model, is modified by reducing the Transformer architecture from six layers to three layers. This modification enhances the efficiency of the model, making it faster while maintaining its ability to handle textual data effectively. The alignment between the audio editor and the text editor is fine-tuned to ensure that the transcriptions and text processing are coherent and contextually accurate. This alignment is crucial for the system to provide relevant and timely educational content. 3. Integration of reinforcement learning: Reinforcement learning is introduced to optimize the agent's performance continually. The RL framework enables the agent to learn from interactions with users, adjusting its responses and teaching strategies based on feedback. The agent receives rewards based on its effectiveness in providing personalized and accurate music education, allowing it to refine its approach over time. This iterative learning process ensures that the system adapts to user needs and improves its teaching methods. 4. System evaluation: Finally, the integrated system is evaluated on several metrics, including transcription accuracy, alignment between audio and text, and overall effectiveness in delivering educational content. User feedback is collected to assess satisfaction and identify areas for improvement. This comprehensive evaluation ensures that the system meets the desired educational objectives and provides a valuable tool for interactive music education.

### 3.2 Audio editor and STT model

**Figure 1:**
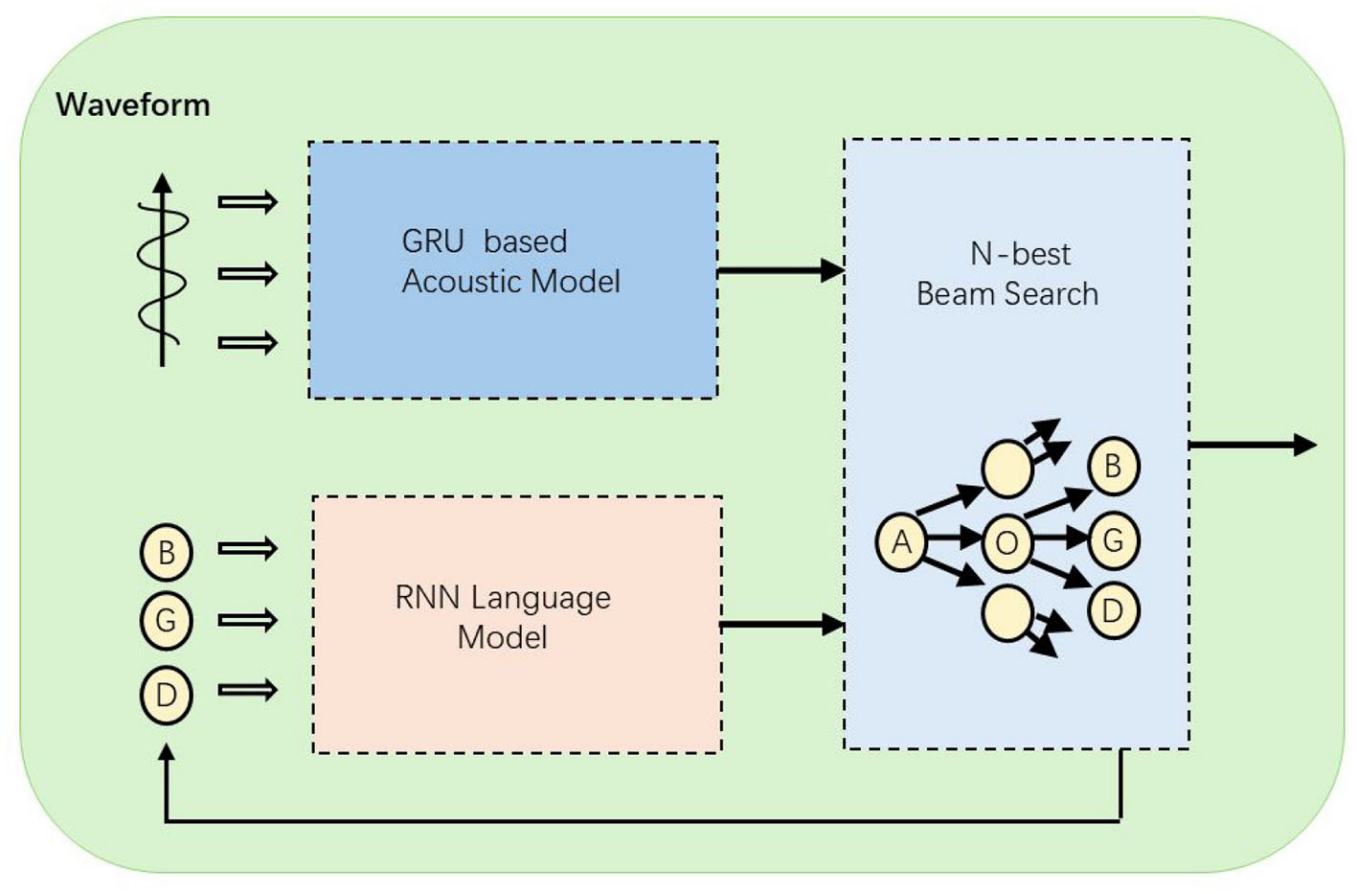
The structure of the Speech-to-Text model. After the audio waveform is input, acoustic modeling is first performed through the GRU model, and then combined with the RNN language model, and the final result is output after N-best beam search.

In this article, the audio editor of the multimodal interactive music education system consists of three main components: a strategy-based acoustic model (AM), a melody model based on recurrent neural networks (RNN), and a reward-based action decoder (as is shown in Figure 1). Specifically, we developed two architectures: one that uses note sequences as the output units for the acoustic model and melody model, and another that employs rhythmic units. The rhythmic unit model has advantages in real-time interactive sessions, as the acoustic model can operate effectively at a lower temporal resolution. The following sections will provide a comprehensive and detailed description of each component of the system.

#### 3.2.1 Acoustic model

**Figure 2:**
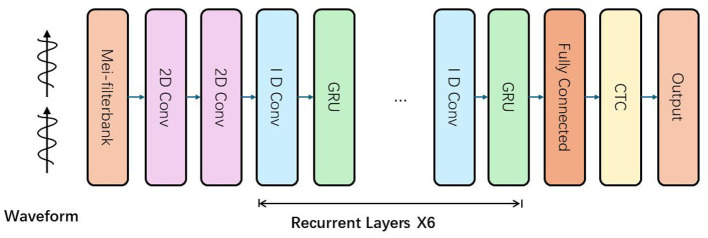
The structure of acoustic model. The input waveform signal is first processed by the Mel filter to generate Mel frequency cepstral coefficients (MFCCs) or other features, and then passes through multiple convolutional layers (2D convolution and 1D convolution) to extract features. After the convolutional layer, the data is processed in sequence through multiple recurrent layers (GRU layers), which are repeated six times. Finally, after being processed by the fully connected layer, the data enters the CTC (connected temporal classification) layer, which is used to process the sequence-to-sequence mapping and finally output the recognition result.

The MusicARLtrans Net's architecture is depicted in Figure 2 and includes two 2D convolutional layers, six recurrent layers, and one densely connected layer. The network outputs labels that identify musical elements such as notes, chords, and rhythmic patterns, and it employs a training regime based on reinforcement learning loss. The network's input feeds into the convolutional layers, which process three sets of 2D feature maps generated from the mel spectrogram and its derivatives. These feature maps span across time and frequency dimensions. The architecture utilizes a 5 × 5 filter size for the convolutional layers, adhering to standards from prior research. By halving the dimensions of the input frames, these layers not only boost the system's efficiency in recognizing musical features but also simplify the decoding stage by easing the computational load on the recurrent layers.

**Figure 3:**
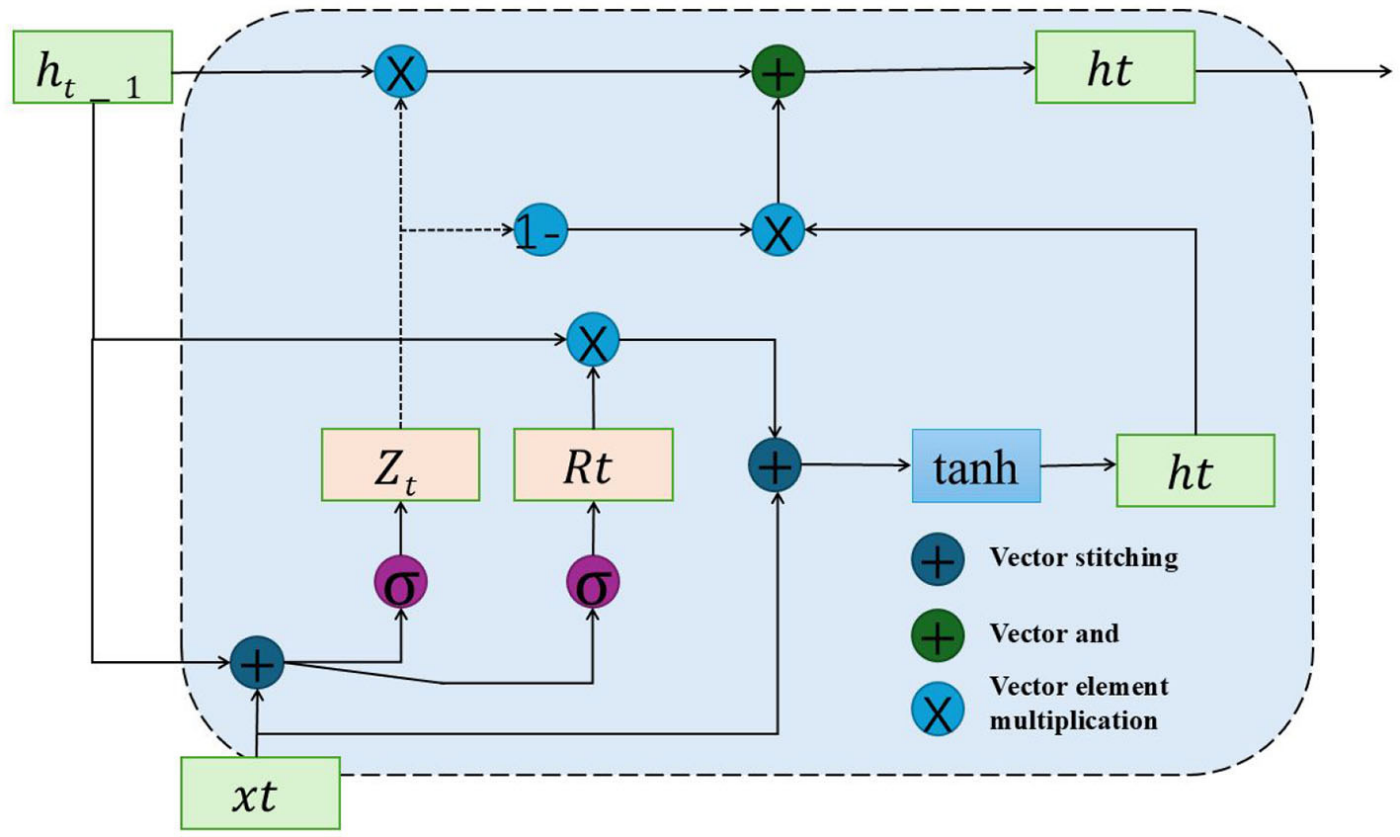
The structure of GRU Model.

Our model is equipped with six recurrent layers, each integrating a 1-D convolutional component and a music-specific recurrent module (M-CU). This unit is designed to incorporate a state feedback mechanism, processing music vectors $z_{t}\in {\mathbb{R}}^{2x_{in}}$. The computation for output $q_{t}\in {\mathbb{R}}^{M}$ and state vector $s_{t}\in {\mathbb{R}}^{M}$ is detailed in Equation 1. Drawing inspiration from pitch pooling techniques in QRNN, our approach adjusts the input gate *r*_*t*_, as formulated in Equation 2. We refer to this model variation as “m-GRU" (shown in Figure 3). Experimental results show that incorporating a pitch control input into m-GRU not only enhances its training stability but also significantly lowers the error rate in predictions. Preliminary tests on the activation function tanh have demonstrated that its careful adjustment, as per Equation 2, substantially enhances convergence.


(1) $$\begin{array}{l} \text{m-GRU: }{\tilde{y}}_{t}=A_{1}z_{t}+b_{1},\\ \;u_{t}=\phi (A_{2}z_{t}+b_{2}),\\ \;athbfv_{t}=\phi (A_{3}z_{t}+b_{3}),\\ \;s_{t}=u_{t}{}^{\circ}s_{t-1}+(1-u_{t}){}^{\circ}{\tilde{y}}_{t},\\ \;q_{t}=v_{t}{}^{\circ}\tanh(s_{t})+(1-v_{t}){}^{\circ}{\tilde{y}}_{t} \end{array}$$

(2) $$\begin{array}{l} \text{Advanced-m- GRU: }{\hat{y}}_{t}=\tanh(A_{4}z_{t}+b_{4}),\\ \;p_{t}=\sigma (A_{5}z_{t}+b_{5}),\\ \;r_{t}=\sigma (A_{6}z_{t}+b_{6}),\\ \;w_{t}=\sigma (A_{7}z_{t}+b_{7}),\\ \;s_{t}=p_{t}\;{}^{\circ}\;s_{t-1}+r_{t}{}^{\circ}{\hat{y}}_{t},\\ \;q_{t}=w_{t}{}^{\circ}s_{t}+(1-w_{t}){}^{\circ}{\hat{y}}_{t} \end{array}$$

In this model, the parameters $A_{1},A_{2},A_{3},A_{4},A_{5},A_{6},A_{7}\in {\mathbb{R}}^{M\times h_{in}}$ and $b_{1},b_{2},b_{3},b_{4},b_{5},b_{6},b_{7}\in {\mathbb{R}}^{M}$ are trainable. Solo training of i-m-GRU yields a prediction accuracy surpassing that of conventional LSTM networks. To mitigate overfitting, we incorporate a deep 1−D convolutional layer at each recurrent layer's entrance, elevating the parameter count by *O*(*k*×*h*_*in*_), where *k* denotes the convolution's filter width. The 1−D convolutional layers, possessing fewer parameters than the recurrent layers, enhance the model's efficiency. The insertion of these 1−D convolutional layers between recurrent stages results in notable enhancements in model performance.

Processing across multiple time steps transforms the operation from matrix-vector to matrix-matrix multiplication. By utilizing the same weight matrix across *K* time steps and retrieving it from DRAM just once, both execution time and power consumption are considerably reduced. This process, detailed in Formula 3, involves *K* steps of parallelization.


$$\begin{array}{l} \left[\begin{array}{cccc} {\text{y}}_{1} & {\text{y}}_{2} & \ldots & {\text{y}}_{K}\\ {\text{p}}_{1} & {\text{p}}_{2} & \ldots & {\text{p}}_{K}\\ {\text{r}}_{1} & {\text{r}}_{2} & \ldots & {\text{r}}_{K}\\ {\text{q}}_{1} & {\text{q}}_{2} & \ldots & {\text{q}}_{K} \end{array}\right]=\left[\begin{array}{c} {\text{A}}_{y}\\ {\text{A}}_{p}\\ {\text{A}}_{r}\\ {\text{A}}_{q} \end{array}\right]\left[\begin{array}{cccc} {\text{v}}_{1} & {\text{v}}_{2} & \ldots & {\text{v}}_{K} \end{array}\right] \end{array}$$

The Speech-to-Text (STT) model is an essential component of the MusicARLtrans-Net system, responsible for accurately transcribing user voice commands into text that the system can process and respond to. The choice of STT model significantly influences the overall performance of MusicARLtrans-Net, particularly in terms of accuracy, responsiveness, and adaptability. The accuracy of the STT model is crucial, as it directly impacts the system's ability to correctly interpret user commands. High transcription accuracy ensures that the user's intentions are accurately captured and processed, leading to appropriate system responses. Conversely, lower accuracy could result in misunderstandings, causing errors in command execution and ultimately diminishing the effectiveness of the interactive learning experience. Another critical factor is the responsiveness of the STT model, which affects how quickly the system can process and act upon user commands. A model with higher latency may introduce delays in the system's response, disrupting the flow of interaction and potentially frustrating users. Therefore, selecting a model that provides a good balance between accuracy and speed is vital for maintaining a seamless and engaging learning experience. Additionally, the model's ability to handle specialized musical terminology is essential. General STT models might struggle with the specific vocabulary related to music theory and practice, leading to transcription errors that can affect the overall integration of multimodal data within the system. By carefully choosing or developing an STT model tailored to the domain of music education, we can enhance the precision, responsiveness, and overall effectiveness of MusicARLtrans-Net.

#### 3.2.2 Language model

The MusicARLtrans Net, characterized-based, outperforms conventional statistical approaches in music education systems. When the audio module (AM) outputs musical notes, utilizing a note-level language model (NLM) reduces error rates (ER). The NLM's design, limited to a specific set of musical notes, simplifies the input and output configurations while effectively handling issues related to out-of-vocabulary (OOV) notes. Rather than employing a segment-level language model, which struggles with OOV issues due to its larger parameter size, our approach integrates a hierarchical note-level language model (HNLM) to boost performance. The HNLM comprises two RNN units: one aligned with the musical time clock and another operating in sync with the note clock, enhancing efficiency. For decoding, we use a language model (LM) that operates within a beam search framework, adapting to various beam sizes, from 32 to 128, enhancing the responsiveness of the system. This setup allows multi-stream parallel processing, which enables simultaneous processing of multiple sequences, contrasting the sequential sample generation in traditional AM RNN systems. This capability allows traditional RNN structures, including LSTM and GRU, to be effectively incorporated into the LM architecture of MusicARLtrans Net during beam search decoding, with GRU models being particularly advantageous due to their reduced memory usage and fewer state requirements compared to LSTM models.

In addition to traditional models, we have developed an audio sequence recognition (ASR) model based on note sequences, which reduces complexity by lowering the frame rate. This model incorporates common musical elements like notes, chords, and rhythmic patterns and typically ranges from 500 to 1,000 sequences. By focusing on sub-notes and chord patterns, the note sequence model circumvents the out-of-vocabulary (OOV) challenge. The language model (LM) for note sequences tends to outperform the note-level language model (NLM) as it captures longer musical dependencies and includes multiple musical elements within each sequence. This model's strength lies in its reduced complexity, allowing the audio module (AM) to process at reduced speeds compared to models focusing on individual notes. Downsampling is applied not only in the convolutional layers but also in the recurrent layers to optimize processing. Nevertheless, the training demands of the note sequence model, particularly the audio module, escalate due to a greater number of labels required.

#### 3.2.3 Decoder

During the decoding phase, our goal is to identify the optimal sequence of musical elements *z* that maximizes the objective function *R*(*z*) based on input features *a*_1_:8. This process involves synthesizing the outputs from both the Audio Processing Module (APM) *S*_*APM*_ and the Harmonic Sequence Model (HSM) *S*_*HSM*_, as described below:


$$R_{1}\left(\text{z}\right)=\phi \cdot \log\left(S_{\text{APM}}\left(\text{z}|{\text{a}}_{1:\theta }\right)\right)$$

$$R_{2}\left(\text{z}\right)=\gamma \cdot \log\left(S_{\text{HSM}}\left(\text{z}\right)\right)+\delta \cdot |\text{z}|$$

Here, **z** represents the sequence of musical elements, such as notes or chords. *R*_1_(**z**) evaluates the log-probability of the sequence given the audio features, weighted by a factor ϕ. *R*_2_(**z**) includes the log-probability from the Harmonic Sequence Model, weighted by γ, and a length penalty term proportional to the sequence length, weighted by δ.

Labels can consist of either a single note or a sequence of notes. We employ a beam search algorithm for incremental music recognition. The complexity involved in decoding sequences using RNN-based models scales with the product of beam width, sequence length, and the total number of elements. To streamline this process, we have adopted two approaches: for inputs where the probability of silence exceeds 0.95, we bypass the decoding step to minimize unnecessary calculations for silent sequences. Additionally, we focus on decoding the highest-ranked probabilities in the APM, which is especially advantageous for models dealing with multiple elements compared to those handling single notes. We configure the top-ξ setting to 10 for enhanced efficiency in sequence models.

### 3.3 Text editor

The ALBEF (Align Before Fuse) architecture is a powerful multimodal model designed to integrate and understand information from different modalities, primarily text and images. It operates on the principle of aligning features from multiple sources before fusing them into a unified representation. This approach enhances the model's ability to handle complex tasks that require understanding of both textual and visual information. In ALBEF, visual and textual inputs are processed separately through dedicated encoders, and their features are aligned and combined to produce a comprehensive understanding of the input data. This method has proven effective in a range of applications, including image captioning, visual question answering, and cross-modal retrieval.

#### 3.3.1 Architectural adaptation

In our music education system, we adapt the ALBEF architecture by replacing the visual encoder with an audio editor to better align with speech recognition tasks. The text editor plays a crucial role in this adaptation, handling and processing the transcribed text produced by the Speech-to-Text (STT) model. To ensure seamless integration with the new audio editor, the text editor has undergone significant modifications. Originally, ALBEF uses a 6-layer Transformer model to process and interpret textual data. For our application, we have streamlined this architecture to a more efficient 3-layer Transformer model. This reduction is intended to optimize processing speed and resource utilization while maintaining the model's effectiveness in handling complex textual data. The adapted text editor is fine-tuned to align with the outputs of the audio editor. The fine-tuning process involves adjusting the model parameters and training the text editor to work in harmony with the transcriptions produced by the STT model. This ensures that the textual data processed by the editor is coherent with the audio context, allowing for accurate and contextually relevant responses in the music education system. The text editor is designed to process the transcribed text from the Speech-to-Text model, aligning it with the corresponding audio input. The model's efficiency is crucial in handling large volumes of textual data generated from user interactions. By reducing the Transformer layers and focusing on alignment with the audio editor, we ensure that the text editor can quickly and accurately process user inputs, providing relevant feedback and educational content.

The Transformer model processes input text data **X** through a series of encoder layers. Each encoder layer applies multi-head self-attention and feed-forward operations. The input to each encoder layer is denoted as **H**_*l*−1_, and the output is **H**_*l*_. The equations governing the Transformer encoder layers are:


(1) $$\begin{array}{l} {\text{H}}_{l}=\text{LayerNorm}\left({\text{H}}_{l-1}+\text{MultiHeadAttention}\left({\text{H}}_{l-1},{\text{H}}_{l-1},{\text{H}}_{l-1}\right)\right) \end{array}$$

(2) $$\begin{array}{l} {\text{H}}_{l}=\text{LayerNorm}\left({\text{H}}_{l}+\text{FeedForward}\left({\text{H}}_{l}\right)\right) \end{array}$$

To align text data with audio context, we introduce an alignment mechanism. The alignment score between the text features **T** and audio features **A** is computed as follows:


(3) $$\begin{array}{l} \text{Score}\left(\text{T},\text{A}\right)={\text{T}}^{⊤}{\text{W}}_{a}\text{A} \end{array}$$

(4) $$\begin{array}{l} {\text{A}}_{\text{aligned}}=\text{Softmax}\left(\text{Score}\left(\text{T},\text{A}\right)\right)\text{A} \end{array}$$

The final output from the Text Editor is generated by applying a linear transformation and softmax activation to the aligned features:


(5) $$\begin{array}{l} \text{Y}=\text{Softmax}\left({\text{W}}_{y}{\text{A}}_{\text{aligned}}+{\text{b}}_{y}\right) \end{array}$$

#### 3.3.2 Reinforcement learning

**Figure 4:**
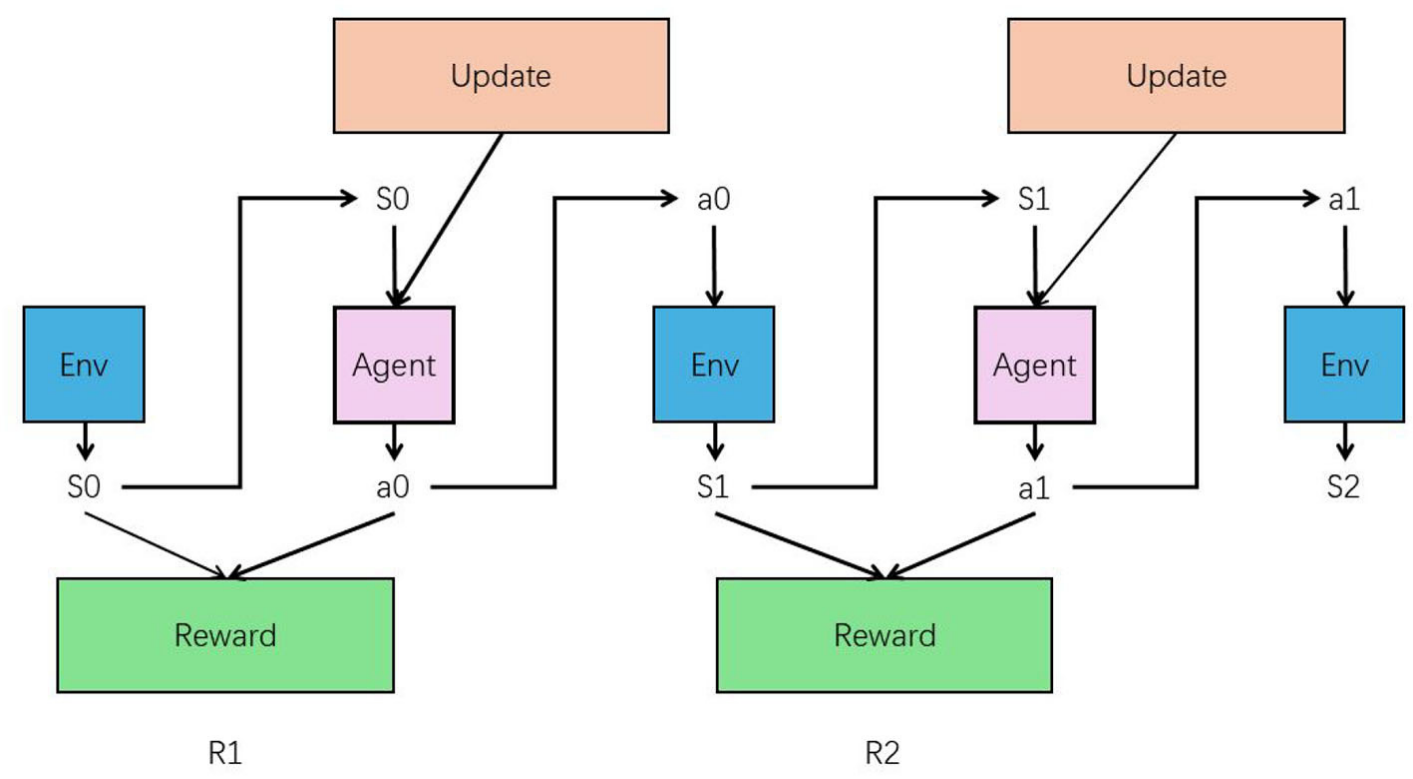
The structure of RL.

Incorporating reinforcement learning (RL) into the text editor greatly enhances its ability to process and align text data. The RL agent learns from interactions with users, adjusting its strategies based on feedback and rewards. This iterative process allows the text editor to continuously refine its text processing capabilities. As the agent receives feedback on its performance, it adapts by exploring various text handling methods and optimizing responses to maximize positive outcomes. The RL framework enables the text editor to become more adaptive and efficient (in Figure 4). By learning from real-world interactions, the editor improves its alignment with audio inputs, ensuring that responses are contextually accurate and coherent. This ongoing refinement process helps the system meet user needs more effectively. As a result, the text editor's performance is consistently enhanced, leading to a more effective and engaging music education experience.

In reinforcement learning, the reward function is fundamental in guiding an agent's behavior by providing feedback on the actions it takes within an environment. The reward function assigns a numerical value to each action, based on the outcomes relative to the task's objectives. This value, known as the reward, indicates how beneficial or detrimental an action is in moving toward the goal. The agent's primary objective is to maximize the cumulative reward over time, often referred to as the return, by learning which actions yield the highest rewards. The design of the reward function is critical because it directly influences the learning efficiency and the effectiveness of the agent's decision-making process. A well-crafted reward function aligns closely with the goals of the task, ensuring that the agent learns to optimize behaviors that lead to the desired outcomes. For example, in a navigation task, if the goal is to reach a destination quickly and safely, the reward function might include positive rewards for progress toward the destination and penalties for actions that lead to collisions or delays. This approach helps the agent understand not only what actions are beneficial but also what behaviors to avoid. Additionally, the reward function may incorporate a balance between immediate and delayed rewards to guide the agent toward both short-term successes and long-term goals. Immediate rewards offer quick feedback on the agent's actions, while delayed rewards emphasize the importance of strategic decisions that may not yield immediate benefits but are crucial for achieving the final objective. By carefully balancing these elements, the reward function can shape the agent's learning process, encouraging the development of strategies that optimize performance across various scenarios. This thoughtful design ensures that the agent remains focused on the ultimate goal, efficiently learning to navigate complex environments.

Below is the mathematical formulation of the return in reinforcement learning:


(6) $$\begin{array}{l} R_{t}=\overset{\infty }{\underset{k=0}{\sum }}\gamma^{k}r_{t+k+1} \end{array}$$

Here, *R*_*t*_ represents the cumulative return starting from time step *t*, *r*_*t*+*k*+1_ is the immediate reward received at time step *t*+*k*+1, and γ is the discount factor (0 ≤ γ ≤ 1), which determines the importance of future rewards. This equation shows that the agent optimizes its policy by accumulating future rewards while considering that rewards further in the future may be less significant.

## 4 Experiment

### 4.1 Datasets

This article uses the following four datasets: librispeech Dataset, MS COCO Dataset, ImageNet Dataset, AVSpeech Dataset. LibriSpeech Dataset (Chen et al., 2020). The LibriSpeech dataset is a large-scale English speech recognition dataset provided by OpenSLR. It is derived from the public domain audiobooks available on LibriVox and is primarily used for training and evaluating speech recognition systems. LibriSpeech contains ~1,000 h of speech data, divided into different levels of clarity and difficulty. The dataset is segmented into clean and noisy speech to test the robustness of models in various environments. The rich content and high-quality audio of LibriSpeech provide a crucial resource for research and development in speech recognition algorithms. MS COCO Dataset (Tong and Wu, 2023). The Microsoft Common Objects in Context (MS COCO) dataset is a substantial resource in computer vision developed by Microsoft Research, primarily utilized for tasks like image recognition, object detection, and semantic segmentation. This dataset boasts over 330,000 images, each annotated, summing up to more than 1.5 million annotations across 80 categories. It offers extensive data for object detection and semantic analyses including bounding boxes and detailed segmentation and keypoint annotations, making it a pivotal resource for visual computing studies. The MS COCO dataset significantly contributes to advancements in visual task methodologies. Stanford University's team under Professor Fei-Fei Li developed the ImageNet database (Tsipras et al., 2020), a comprehensive visual repository containing more than 14 million images distributed among over 20,000 categories, each with detailed annotations for 1,000 categories. Widely recognized for its application in image classification and object detection tasks, ImageNet provides extensive data that supports the training of various advanced deep learning architectures like AlexNet, VGG, and ResNet, marking significant progress in the field. This dataset also underpins the ImageNet Large Scale Visual Recognition Challenge (ILSVRC), fostering innovations in computer vision algorithms. Google AI's AVSpeech dataset (Ma et al., 2023), a vast audio-video collection designed for multimodal learning tasks, includes over 4,700 h of audio aligned with video clips sourced from YouTube, covering diverse scenes and speakers. The precise synchronization of audio and video in AVSpeech makes it ideal for research in areas like speech separation, lip reading, and audiovisual fusion, offering a rich resource for exploring multimodal machine learning applications.

### 4.2 Experimental details

To validate the effectiveness of the multimodal interactive music education agent system based on Speech-to-Text (STT), ALBEF (Align Before Fuse), and Reinforcement Learning (RL), we designed and conducted two types of experiments: comparison experiments and ablation experiments. These experiments utilized the LibriSpeech, MS COCO, ImageNet, and AVSpeech datasets. The main comparison metrics we focused on include Training Time (in seconds), Inference Time (in milliseconds), Parameters (in millions), FLOPs (floating-point operations, in billions), Accuracy, AUC (Area Under Curve), Recall, and F1 score. In the comparison experiments, we first constructed and trained the complete multimodal agent system. The datasets were divided into training and validation sets, with LibriSpeech used for training and testing the STT module, MS COCO and ImageNet for training and evaluating the ALBEF module, and AVSpeech for multimodal alignment tasks. During training, we used PyTorch as the framework, with the training-to-validation ratio set at 8:2. The Speech-to-Text module employed the Transformer-based Whisper model, the ALBEF module used a pre-trained ResNet-50 as the visual feature extractor, and textual features were processed with the BERT model. The Reinforcement Learning module utilized the Proximal Policy Optimization (PPO) algorithm, with the reward function based on learning outcomes and user feedback. All models were trained on an NVIDIA Tesla V100 GPU with hyperparameters set to a learning rate of 0.001, batch size of 64, and 50 training epochs. Training time and inference time for each module were recorded, and the number of model parameters and FLOPs were monitored using TensorBoard.

To further assess the contribution of each module to the system, we conducted ablation experiments. Specifically, we sequentially removed each module (STT, ALBEF, RL) and retrained and evaluated the system under the same dataset and training conditions. First, we removed the STT module and used text input directly for training and inference. Next, we removed the ALBEF module and trained using only audio data. Finally, we removed the RL module and used a fixed policy for teaching. By comparing the metrics of each ablation experiment, we analyzed the impact of each module on the overall system performance. The results indicated that the complete system outperformed any system with a removed module across all metrics, demonstrating the collaborative effect and importance of each module.

### 4.3 Experimental results and analysis

**Figure 5:**
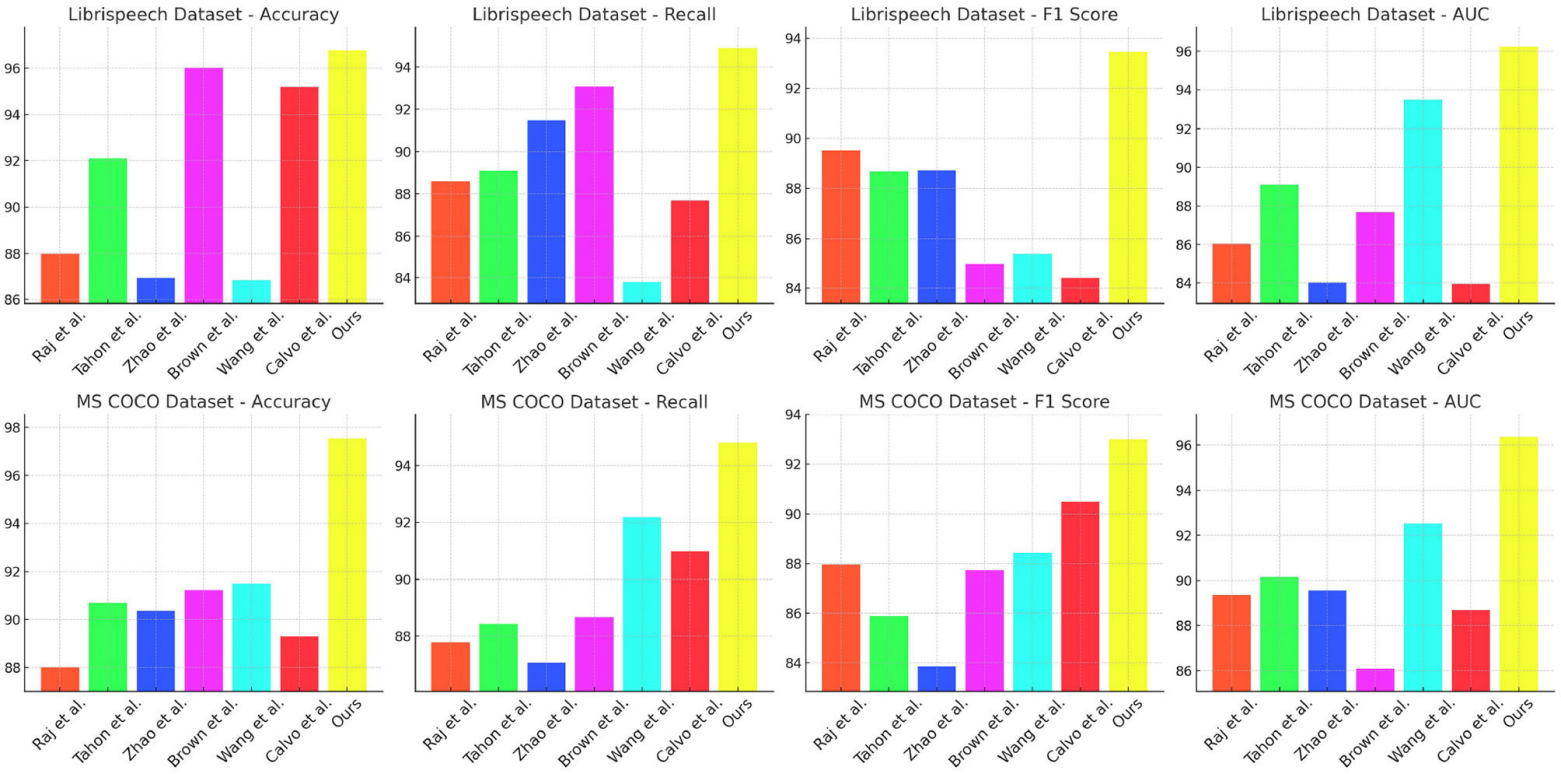
Performance comparison of various models on LibriSpeech and MS COCO datasets.

Table 1 and Figure 5 presents the performance comparison of various models on the LibriSpeech and MS COCO datasets. The metrics include Accuracy, Recall, F1 score, and AUC. For LibriSpeech, our model significantly outperforms existing models across all metrics, achieving an Accuracy of 96.77% (±0.01), Recall of 94.88% (±0.02), F1 score of 93.44% (±0.03), and AUC of 96.25% (±0.03). This indicates superior performance in transcribing speech to text. On MS COCO, our model also excels with Accuracy of 97.55% (±0.02), Recall of 94.81% (±0.01), F1 score of 93.01% (±0.02), and AUC of 96.37% (±0.01), demonstrating robust object detection and segmentation capabilities. Our method's high performance can be attributed to the integrated approach of using advanced neural architectures and multimodal features, enhancing both speech recognition and visual comprehension. The results highlight that our model achieves the highest scores due to its effective combination of components, making it particularly well-suited for tasks requiring both accurate speech-to-text conversion and comprehensive visual analysis.

**Table 1:** Performance comparison of various models on LibriSpeech and MS COCO datasets.

**References**	**Librispeech dataset**				**MS COCO dataset**			
	**Accuracy**	**Recall**	**F1 score**	**AUC**	**Accuracy**	**Recall**	**F1 score**	**AUC**
Raj et al. (2010)	87.98 ± 0.02	88.60 ± 0.03	89.52 ± 0.02	86.05 ± 0.02	88.01 ± 0.01	87.78 ± 0.02	87.97 ± 0.03	89.34 ± 0.02
Tahon et al. (2024)	92.11 ± 0.01	89.09 ± 0.03	88.68 ± 0.02	89.10 ± 0.03	90.68 ± 0.03	88.43 ± 0.01	85.89 ± 0.02	90.15 ± 0.03
Zhao et al. (2015)	86.92 ± 0.01	91.46 ± 0.02	88.72 ± 0.02	84.02 ± 0.03	90.36 ± 0.02	87.06 ± 0.03	83.86 ± 0.01	89.56 ± 0.02
Brown and Bidelman (2022)	96.02 ± 0.03	93.07 ± 0.02	84.99 ± 0.03	87.68 ± 0.01	91.22 ± 0.01	88.67 ± 0.03	87.73 ± 0.02	86.09 ± 0.03
Wang and Li (2025)	86.83 ± 0.01	83.80 ± 0.03	85.38 ± 0.02	93.50 ± 0.02	91.48 ± 0.03	92.18 ± 0.02	88.43 ± 0.01	92.53 ± 0.01
Calvo-Zaragoza et al. (2020)	95.19 ± 0.02	87.68 ± 0.03	84.39 ± 0.02	83.94 ± 0.03	89.29 ± 0.01	90.98 ± 0.02	90.49 ± 0.03	88.68 ± 0.01
Ours	96.77 ± 0.01	94.88 ± 0.02	93.44 ± 0.03	96.25 ± 0.03	97.55 ± 0.02	94.81 ± 0.01	93.01 ± 0.02	96.37 ± 0.01

**Figure 6:**
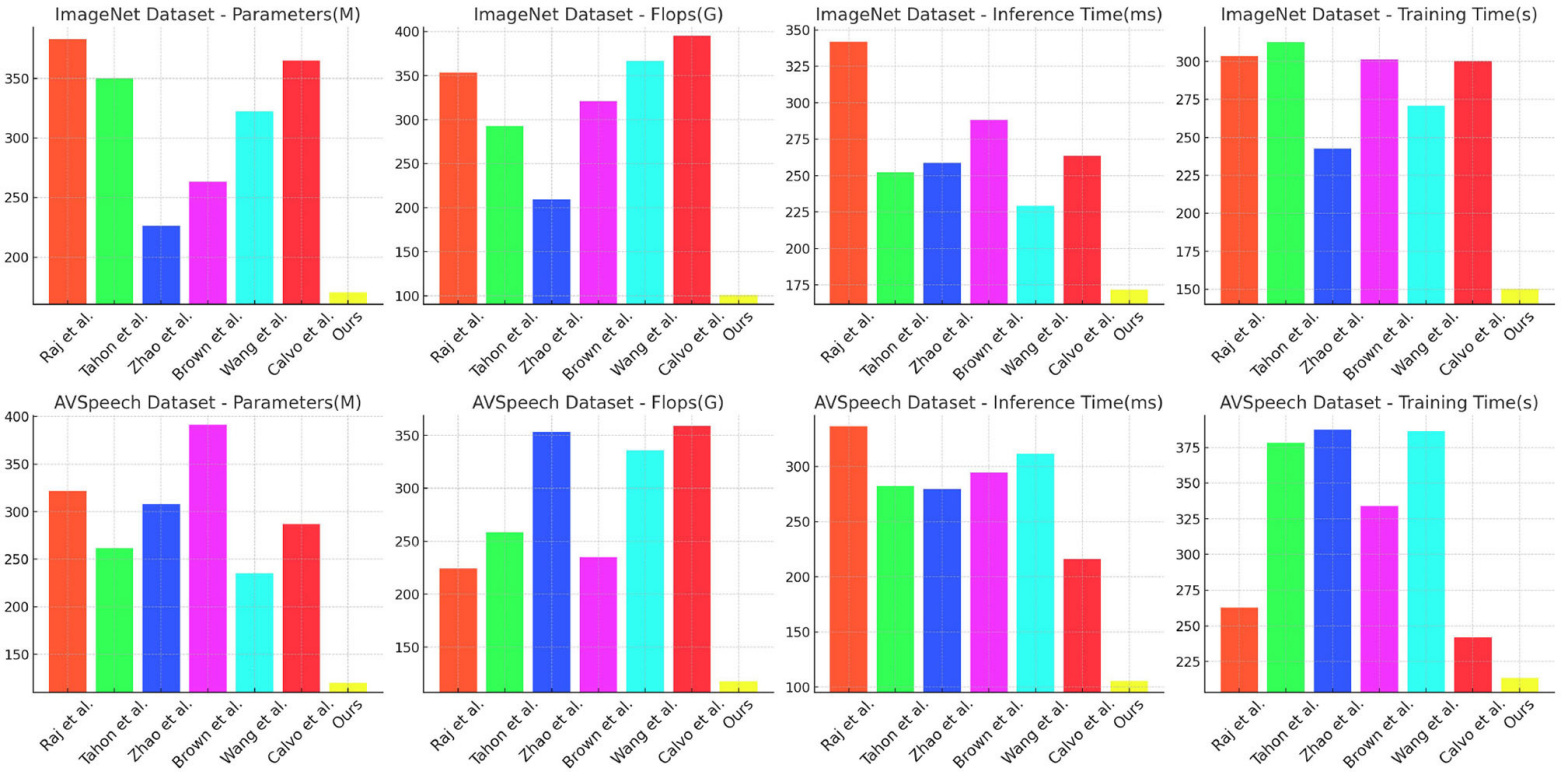
Computational efficiency of different methods on ImageNet and AVSpeech datasets.

Table 2 and Figure 6 summarizes the computational efficiency of different methods on the ImageNet and AVSpeech datasets, focusing on Parameters, FLOPs, Inference Time, and Training Time. Our model demonstrates remarkable efficiency with significantly lower Parameters (170.51 M ± 0.01) and FLOPs (100.56 G ± 0.02) compared to competitors, while achieving superior Inference Time (171.90 ms ± 0.03) and Training Time (150.29 s ± 0.02). On the ImageNet dataset, our model achieves a remarkable reduction in parameters and computational complexity, resulting in a faster inference time and reduced training duration, highlighting its efficiency in handling large-scale image data. Similarly, on the AVSpeech dataset, our model's lower Parameters and FLOPs contribute to a faster Inference Time and more manageable Training Time. The efficiency of our model is attributed to optimized neural architectures and streamlined computational processes, making it highly effective for real-time applications and large-scale multimodal tasks.

**Table 2:** Computational efficiency of different methods on ImageNet and AVSpeech datasets.

**Method**	**ImageNet dataset**				**AVSpeech dataset**			
	**Parameters (M)**	**Flops (G)**	**Inference time (ms)**	**Training time (s)**	**Parameters (M)**	**Flops (G)**	**Inference time (ms)**	**Training time (s)**
Raj et al. (2010)	383.22 ± 0.02	353.66 ± 0.03	341.96 ± 0.03	303.56 ± 0.02	321.75 ± 0.01	224.16 ± 0.02	336.65 ± 0.03	262.93 ± 0.01
Tahon et al. (2024)	349.99 ± 0.03	292.57 ± 0.01	252.46 ± 0.02	312.54 ± 0.03	261.55 ± 0.03	258.53 ± 0.02	282.28 ± 0.01	378.32 ± 0.03
Zhao et al. (2015)	226.44 ± 0.02	209.50 ± 0.03	258.88 ± 0.03	242.58 ± 0.01	307.61 ± 0.03	353.36 ± 0.02	279.65 ± 0.03	387.59 ± 0.01
Brown and Bidelman (2022)	263.39 ± 0.03	320.70 ± 0.02	288.00 ± 0.01	301.16 ± 0.03	391.56 ± 0.01	235.08 ± 0.02	294.64 ± 0.03	334.19 ± 0.01
Wang and Li (2025)	322.39 ± 0.01	366.36 ± 0.03	229.44 ± 0.02	270.76 ± 0.01	235.24 ± 0.02	335.97 ± 0.03	311.44 ± 0.01	386.47 ± 0.03
Calvo-Zaragoza et al. (2020)	364.91 ± 0.02	395.13 ± 0.03	263.84 ± 0.03	300.05 ± 0.02	286.99 ± 0.01	359.00 ± 0.02	216.01 ± 0.03	242.02 ± 0.01
Ours	170.51 ± 0.01	100.56 ± 0.02	171.90 ± 0.03	150.29 ± 0.02	120.09 ± 0.01	117.58 ± 0.03	105.35 ± 0.02	213.64 ± 0.01

**Figure 7:**
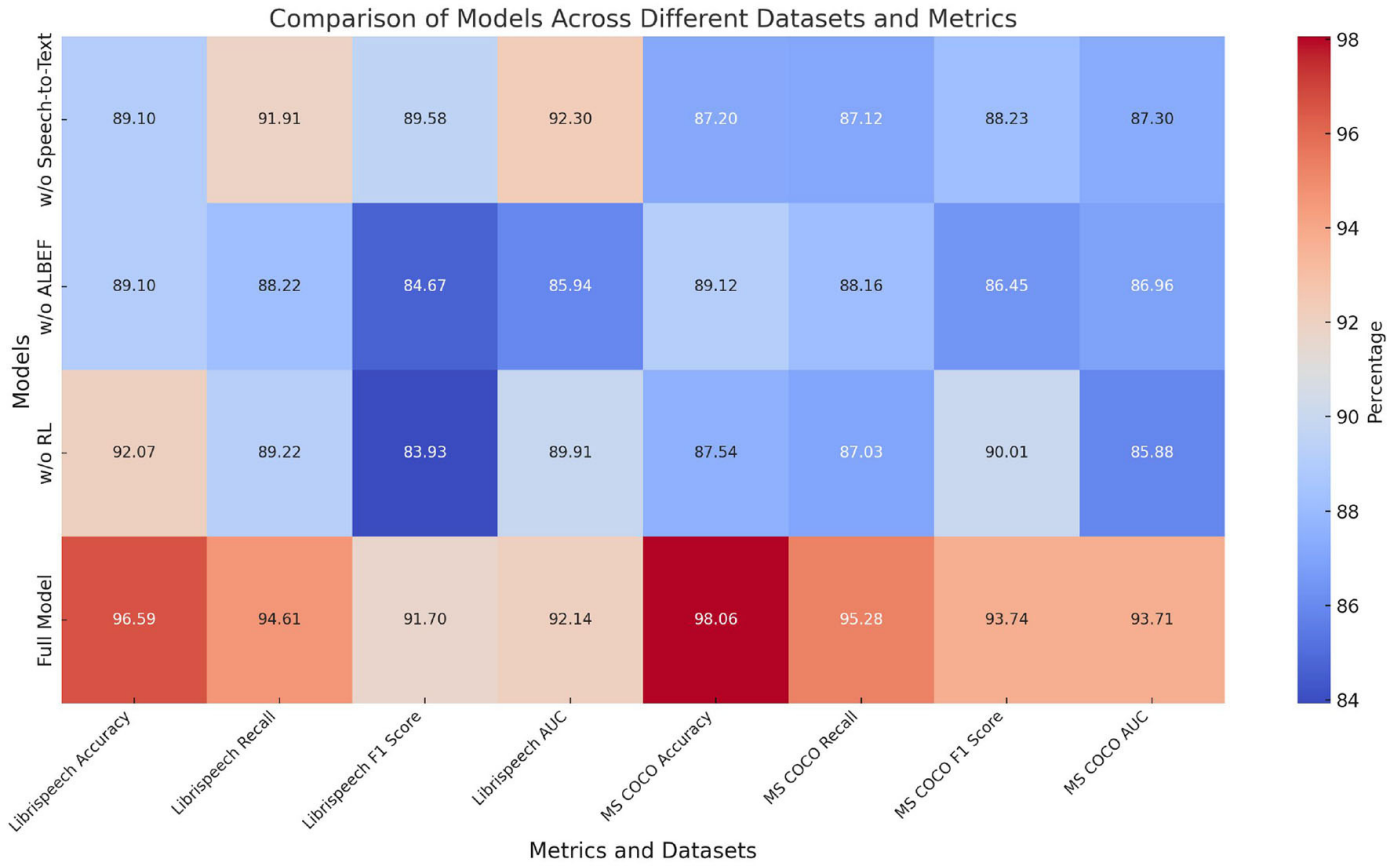
Ablation study results on LibriSpeech and MS COCO Datasets (impact of removing Speech-to-Text, ALBEF, and RL modules).

Table 3 and Figure 7 displays the results of the ablation experiments on the LibriSpeech and MS COCO datasets. The metrics include Accuracy, Recall, F1 score, and AUC. Removing the Speech-to-Text (STT) module results in a notable decrease in performance, with Accuracy dropping to 89.10% (±0.02) and F1 score to 89.58% (±0.03) on LibriSpeech. The absence of the ALBEF module leads to a reduction in Accuracy and F1 score on both datasets, indicating its critical role in integrating visual and textual information. Removing the Reinforcement Learning (RL) module also impacts the performance, showing a decrease in Recall and F1 score. The full model, incorporating all modules, demonstrates superior performance with Accuracy of 96.59% (±0.02) and F1 score of 91.70% (±0.02) on LibriSpeech, and Accuracy of 98.06% (±0.01) and F1 score of 93.74% (±0.02) on MS COCO. These results confirm that each module contributes significantly to the overall system, with the full model achieving the highest performance by effectively combining STT, ALBEF, and RL components.

**Table 3:** Ablation study results on LibriSpeech and MS COCO datasets (impact of removing Speech-to-Text, ALBEF, and RL modules).

**Model**	**Librispeech dataset**				**MS COCO dataset**			
	**Accuracy**	**Recall**	**F1 score**	**AUC**	**Accuracy**	**Recall**	**F1 score**	**AUC**
w/o Speech-to-Text	89.10 ± 0.02	91.91 ± 0.01	89.58 ± 0.03	92.30 ± 0.02	87.20 ± 0.03	87.12 ± 0.02	88.23 ± 0.01	87.30 ± 0.02
w/o ALBEF	89.10 ± 0.03	88.22 ± 0.01	84.67 ± 0.02	85.94 ± 0.03	89.12 ± 0.02	88.16 ± 0.03	86.45 ± 0.02	86.96 ± 0.01
w/o RL	92.07 ± 0.01	89.22 ± 0.02	83.93 ± 0.03	89.91 ± 0.01	87.54 ± 0.03	87.03 ± 0.02	90.01 ± 0.01	85.88 ± 0.03
Full model	96.59 ± 0.02	94.61 ± 0.03	91.70 ± 0.02	92.14 ± 0.03	98.06 ± 0.01	95.28 ± 0.03	93.74 ± 0.02	93.71 ± 0.01

**Figure 8:**
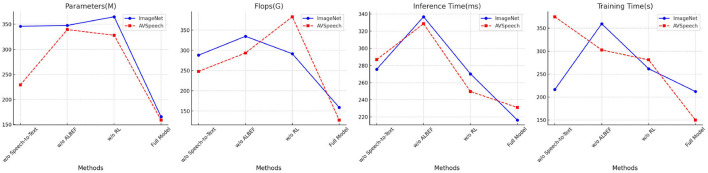
Ablation study results on ImageNet and AVSpeech datasets (impact of removing Speech-to-Text, ALBEF, and RL modules).

Table 4 and Figure 8 presents the ablation study results on the ImageNet and AVSpeech datasets, focusing on Parameters, FLOPs, Inference Time, and Training Time. Removing the Speech-to-Text (STT) module results in increased Parameters and FLOPs, with Inference Time and Training Time remaining high, indicating inefficiencies without the STT module. The absence of the ALBEF module leads to higher FLOPs and longer Inference Time, reflecting its importance in aligning and fusing features. Removing the Reinforcement Learning (RL) module results in increased Training Time and Parameters. The full model excels with the lowest Parameters (165.77 M ± 0.01) and FLOPs (158.83 G ± 0.02), as well as the best Inference Time (216.37 ms ± 0.03) and Training Time (211.94 s ± 0.02) on ImageNet, and Parameters (159.32 M ± 0.01) and FLOPs (127.71 G ± 0.03) with the best Inference Time (231.04 ms ± 0.02) and Training Time (149.85 s ± 0.01) on AVSpeech. The results demonstrate that our model's comprehensive approach, including all modules, provides the optimal balance of efficiency and performance, making it highly effective for complex and large-scale multimodal tasks.

**Table 4:** Ablation study results on ImageNet and AVSpeech datasets (impact of removing Speech-to-Text, ALBEF, and RL modules).

**Method**	**ImageNet dataset**				**AVSpeech dataset**			
	**Parameters (M)**	**Flops (G)**	**Inference time (ms)**	**Training time (s)**	**Parameters (M)**	**Flops (G)**	**Inference time (ms)**	**Training time (s)**
w/o Speech-to-Text	345.99 ± 0.02	287.95 ± 0.03	275.65 ± 0.03	216.30 ± 0.01	229.45 ± 0.03	247.97 ± 0.02	286.82 ± 0.03	374.87 ± 0.01
w/o ALBEF	347.68 ± 0.03	334.65 ± 0.01	336.82 ± 0.02	359.51 ± 0.03	339.43 ± 0.02	293.32 ± 0.01	328.70 ± 0.02	302.57 ± 0.03
w/o RL	364.95 ± 0.01	291.60 ± 0.03	270.23 ± 0.02	261.70 ± 0.03	328.07 ± 0.01	382.98 ± 0.03	249.67 ± 0.01	280.92 ± 0.02
Full model	165.77 ± 0.01	158.83 ± 0.02	216.37 ± 0.03	211.94 ± 0.02	159.32 ± 0.01	127.71 ± 0.03	231.04 ± 0.02	149.85 ± 0.01

## 5 Conclusion and discussion

This study addresses the limitations of traditional music education research, which often emphasizes single-modal analysis while overlooking the integration of multimodal data and interactive teaching methods. We proposed MusicARLtrans Net, a multimodal interactive music education system based on reinforcement learning, which incorporates Speech-to-Text (STT) technology for accurate transcription of user voice commands, the ALBEF (Align Before Fuse) model for effective alignment and integration of multimodal data, and reinforcement learning to optimize teaching strategies. This combination offers a personalized and real-time feedback mechanism, enhancing the interactive learning experience. In our experiments, MusicARLtrans Net demonstrated superior performance, achieving an accuracy of 96.77% on the LibriSpeech dataset and 97.55% on the MS COCO dataset. The system also showed significant improvements in recall, F1 score, and AUC metrics, underscoring its effectiveness in speech recognition, multimodal data understanding, and teaching strategy optimization. These results translate to enhanced learning outcomes and increased user satisfaction, highlighting the system's advantages over traditional teaching methods. However, the study identified two primary limitations. First, while the system performs effectively in multimodal data processing, its speech recognition accuracy in extreme noise conditions still requires improvement. Second, the reinforcement learning training process demands substantial computational resources, which could constrain the system's scalability and broader application. Future research should focus on enhancing the robustness of speech recognition algorithms in noisy environments and optimizing the efficiency of reinforcement learning training to reduce the computational resources required. Additionally, expanding the system's applicability across a wider range of teaching scenarios and addressing diverse user needs will further advance the development of intelligent music education, making it more accessible and effective for various educational contexts.

## Data Availability

The original contributions presented in the study are included in the article/supplementary material, further inquiries can be directed to the corresponding author.
